# Development and Validation of a LC-MS/MS Method for the Simultaneous Estimation of Amlodipine and Valsartan in Human Plasma: Application to a Bioequivalence Study

**DOI:** 10.3797/scipharm.1402-11

**Published:** 2014-03-26

**Authors:** Hemanth Jangala, Poonam Vats, Arshad Hussain Khuroo, Tausif Monif

**Affiliations:** Clinical Pharmacology and Pharmacokinetics, Ranbaxy Research Laboratories, Gurgaon, India.

**Keywords:** Amlodipine, Valsartan, Bioequivalence, Liquid chromatography-mass spectrometry, Non compartmental pharmacokinetics, Solid phase extraction

## Abstract

A reliable, simple, and robust liquid chromatography-tandem mass spectro-metric (LC-MS/MS) method has been developed and validated that employs solid-phase extraction for the simultaneous estimation of amlodipine and valsartan in human K_3_EDTA plasma using amlodipine-d4 and valsartan-d9 as internal standards. Chromatographic separation of amlodipine and valsartan was achieved on the Luna C18 (2)100A (150 × 4.6 mm, 5 μm) column using acetonitrile: 5 mM ammonium formate solution (80:20, v/v) as the mobile phase at a flow rate of 0.8 mL/min in isocratic mode. Quantification was achieved using an electrospray ion interface operating in positive mode, under multiple reaction monitoring (MRM) conditions. The assay was found to be linear over the range of 0.302–20.725 ng/mL for amlodipine and 6.062–18060.792 ng/mL for valsartan. The method has shown good reproducibility, as intra- and interday precisions were within 10% and accuracies were within 8% of nominal values for both analytes. The method was successfully applied for the bioequivalence study of amlodipine and valsartan after oral administration of a fixed dose of the combination. Additionally, as required by the current regulatory bodies, incurred sample reanalysis was performed and found to be acceptable.

## Introduction

Hypertension is a major cause of cardiovascular mortality worldwide. A complex therapeutic regimen is required in hypertensive patients to control blood pressure, which leads to patient discomfort. Patient compliance can be increased by administering fixeddose combinations of antihypertensive agents which effectively lower blood pressure. Several fixed-dose combinations of antihypertensive agents are available on the market. The combination of a calcium channel blocker and an angiotensin II receptor (AT1) blocker as a single-pill, fixed-dose treatment is possibly the best therapy for preventing hypertension [1, 2]. The single pill combination of amlodipine and valsartan, under the said category, is associated with greater absolute blood pressure reductions and fewer dosedependent adverse effects when compared with the use of an individual drug [3]. So, development of analytical methods which simultaneously determine the analytes of a fixed-dose combination has gained more significance. Amlodipine is chemically (±)-3-ethyl 5-methyl 2-[(2-aminoethoxy)methyl]-4-(2-chlorophenyl)-6-methyl-1,4-dihydropyridine-3,5-dicarboxylate, a long-acting calcium channel blocker used in the treatment of hypertension and angina. More than 90% of administered amlodipine gets absorbed when taken orally. It has a half-life of 35–45 hr due to the high volume of distribution (21 L/kg) and gets excreted by hepatic metabolism [4]. Valsartan is chemically *N*-pentanoyl-*N*-{[2'-(1*H*-tetrazol-5-yl)biphenyl-4-yl]methyl}-L-valine, a specific angiotensin II receptor blocker acting on the AT1 receptor subtype. The absolute bioavailability of valsartan is about 23%. Of the absorbed drug, 94–97% is bound to plasma proteins with a half-life of 7–8 h and is excreted in bile [5]. The overall aim was to develop a fast, sensitive, and robust LC-MS/MS method for the simultaneous estimation of amlodipine and valsartan in human plasma and extend its application to assess the bioequivalence of fixed-dose formulations in healthy subjects. Several analytical methods have been reported for the individual determination of amlodipine [6–15] and valsartan [16–19] in biological samples. The methods for the simultaneous determination include capillary electrophoresis [20] and high-performance liquid chromatography (HPLC) with ultraviolet detection [21–24] which were proven to be insensitive for therapeutic drug monitoring. Use of tandem mass spectrometry (LC-MS/MS) had overcome this problem [25–27]. But, the stated methods had longer chromatographic run times which are of limited use from the industrial point of view. Kristoffersen *et al*., 2007, reported a method for the determination of cardiovascular drugs in post-mortem whole blood samples with a run time of 18 min and limit of quantification of approximately 54 ng/mL and 400 ng/mL for amlodipine and valsartan, respectively. Gonzalez *et al*., 2011, reported a method for the quantitation of 55 compounds prescribed in combined cardiovascular therapy with a run time of 18 min and limit of quantification of approximately 0.5 ng/mL and 25 ng/mL for amlodipine and valsartan, respectively. Ramani *et al*., 2009, reported a method using the liquid-liquid extraction technique that quantified simvastatin acid, amlodipine, and valsartan using a single internal standard and applied it to a pharmacokinetic study following the oral administration of valsartan. However, none of these reported methods could suffice the overall requirement of the method.

The developed method offers the advantages of a simple sample preparation procedure without the matrix effect and charge competition, which are critical challenges in LC-MS/MS method development. The solid-phase extraction procedure, which was employed using the centrifugation technique, offers high throughput with uniformity in extraction. The method had a total analysis time of 4.5 min, which is favored in industries to analyze the samples on a large scale. The use of deuterated internal standards compensated for the variability in sample extraction and LC-MS/MS analysis due to its nearly identical chemical and physical properties to the analytes of interest. The reliability of the method was further proven by performing incurred sample reanalysis, in which the values of the reanalyzed concentration were the same as the original concentration. The wide calibration curve range of the method extends its applicability to all available formulations and strengths of amlodipine and valsartan.

## Results and Discussion

### Optimization of MS Parameters and Chromatographic Conditions

**Fig. 1.**
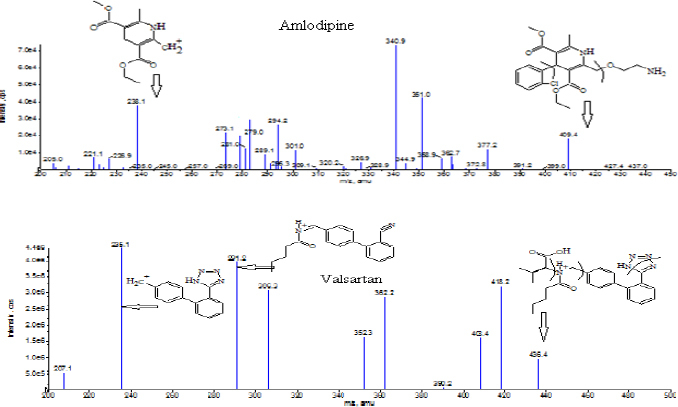
Product ion scans of amlodipine and valsartan, respectively

Mass spectrometric detection was carried out on an API 4000 triple quadrupole instrument equipped with an ESI source operated in the positive ion mode. The ESI source in positive ion mode was selected, as it increased the signal-to-noise ratio of the analytes, which helped in attaining the lower LOQ. During the optimization of the mass spectrometric parameters, strong and stable signals of analytes and internal standards were noted and the ion transitions *m/z* 409.2 → 238.1, 413.2 → 238.1 and 436.2 → 291.5, 445.3 → 300.4 were selected for the MRM of amlodipine, amlodipine-d4, valsartan, and valsartan-d9, respectively. The optimization of the source and compound parameters was done by syringe pump infusion of each analyte. The compound parameters were optimized as follows: declustering potential: 40 V, entrance potential: 10 V, collision cell exit potential: 7 V, and collision energy: 15 V for amlodipine and amlodipine-d4 and 16 V for valsartan and valsartan-d9. The source/gas parameters were optimized as follows: curtain gas: 25, collision gas: 5, ion source gas-1: 40, ion source gas-2: 60, ion spray voltage: 5500 V and temperature: 550°C. The product ion scans of amlodipine and valsartan are shown in Figure 1.

Important parameters like pH of the mobile phase, concentration and type of the buffer (ammonium acetate and ammonium formate) solution, percentage and type of the organic modifier (acetonitrile and methanol), different columns (reverse phase columns such as Discovery C18, Synergie MAX RP and Phenomenex Luna), and different flow rates (0.4–1.8 mL/min) were attempted for better sensitivity and better chromatographic separation of amlodipine and valsartan. The separation was found to be affected by increasing the molarity of the ammonium formate buffer and found to be better with acetonitrile as compared to methanol. During method development, charge competition and matrix effect problems were encountered. Trials have shown that the mobile phase, acetonitrile: 5 mM ammonium formate buffer (80:20, *v/v*) at a flow rate of 0.8 mL/min, with the Luna C18 (2) column nullified the problems of charge competition and matrix effects without compromising on the sensitivity, range, and precision of the method.

### Method Validation

**Fig. 2.**
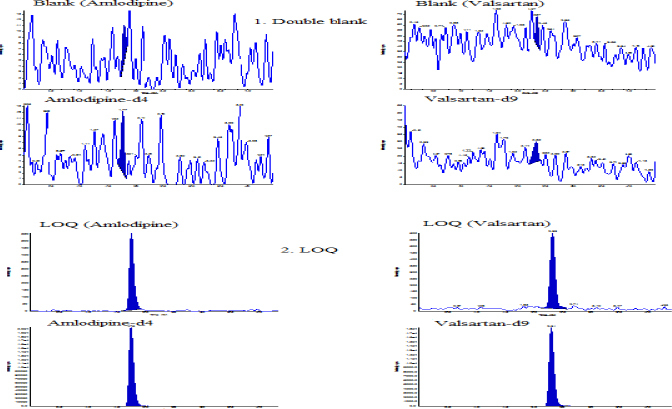
Representative chromatograms of amlodipine and valsartan: 1. double blank sample; 2. LOQ

There was no significant interference observed at the retention times of the analytes and internal standards assessed by calculating % interference derived from the processed blank plasma sample against the mean peak area of the LOQ samples. The typical chromatograms of the double blank and LOQ in human plasma are shown in Figure 2. The limit of quantification was 0.302 ng/mL and 6.062 ng/mL for amlodipine and valsartan, respectively. The precision and accuracy at the LOQ concentration for amlodipine were 4.5% and 99.3% and for valsartan, 8.0% and 103.4%, respectively. The mean signal-to-noise ratios of the LOQ samples with respect to the blank matrix samples were 140.0 and 35.5 for amlodipine and valsartan, respectively. The retention times of amlodipine and valsartan under the optimized chromatographic conditions were 1.7 and 2.2 minutes, respectively.

The calibration curve was shown to be linear for the tested concentration range of both analytes. The mean correlation coefficient of the weighted (1/X^2^ i.e., 1/[concentration]^2^) calibration curves generated in the validation was always > 0.99. Four precision and accuracy batches were run in validation to check intra- and interday precision and accuracy. The results for precision and accuracy are summarized in Table 1. The intraday precision ranged from 1.4 to 4.4% and 2.5 to 9.2% for amlodipine and valsartan, respectively. The interday precision ranged from 1.6 to 4.5% for amlodipine and was 2.9 to 8.0% for valsartan. The intraday accuracy ranged from 93.3 to 108.7% and 95.5 to 103.1% for amlodipine and valsartan, respectively. The interday accuracy ranged from 92.5 to 107.6% for amlodipine and 94.6 to 103.4% for valsartan. Results of the extended precision and accuracy were also found to be acceptable; they endorse the applicability of the method to a bioequivalence study in which many samples are run in longer duration.

**Tab. 1.** Precision and accuracy of the quality control samples of amlodipine and valsartan

Analyte	QC Sample	Accuray^a^		Precision^b^	
		% Intraday^c^	% Interday^d^	% Intraday^c^	% Interday^d^
Amlodipine	LOQQC (0.305ng/mL)	98.5	99.3	4.4	4.5
	LQC (0.887ng/mL)	93.3	92.5	3.5	3.4
	MQC (10.814 ng/mL)	104.9	104.6	1.4	1.6
	HQC (16.3384 ng/mL)	108.7	107.6	1.6	1.8
Valsartan	LOQQC (6.074 ng/mL)	102.6	103.4	9.2	8.0
	LQC (17.864 ng/mL)	103.1	102.3	4.2	4.1
	MQC (6870.877 ng/mL)	102.5	101.5	2.5	2.9
	HQC (14314.328 ng/mL)	95.5	94.6	3.8	3.3

^a^ Expressed as 100x(mean calculated concentration)/(nominal concentration);

^b^ Expressed as 100x(standard deviation of calculated concentration)/ (mean calculated concentration);

^c^ n=12;

^d^ n=24.

The coefficient of variance for the matrix effect samples between 0.9–6.7% and accuracy between 89.6–102.9% indicated that there is no matrix effect in plasma, including the lipemic and hemolyzed batches for both of the analytes. The absolute matrix effect at the LQC, MQC, and HQC level was between 0.97 and 1.02 for both of the analytes. It indicated that there is neither ion-suppression nor ion-enhancement in the developed method. The recoveries of the analytes and ISTD were constant at all of the studied concentration levels. The mean extraction recoveries of amlodipine, valsartan, amlodipine-d4, and valsartan-d9 were 78.7%, 82.6%, 89.3%, and 92.4%, respectively. The CV of mean recovery across the QC levels was < 7% for both analytes. The results, relative matrix effect, AME, and recovery of the method are presented in tables 2 & 3.

**Tab. 2.** Variability in analyte concentration in different lots of human plasma (relative matrix effect)

Plasma lot	Amlodipine		Valsartan	
	LOQQC^a^ (ng/mL)	HQC^a^ (ng/mL)	LOQQC^a^ (ng/mL)	HQC^a^ (ng/mL)
Lot-1	0.301	17.290	5.777	13738.466
Lot-2	0.319	17.172	5.217	13758.835
Lot-3	0.304	17.185	5.266	13795.288
Lot-4	0.312	16.237	5.643	13913.527
Lot-5^d^	0.313	16.274	5.490	13868.357
Lot-6^e^	0.321	16.825	5.665	13791.939
Mean	0.312	16.830	5.510	13811.068
Nominal concentration (ng/mL)	0.304	16.357	6.151	14303.577
%CV^b^	3.8	3.1	6.7	0.9
% Accuracy^c^	102.5	102.9	89.6	96.6

^a^ Mean of duplicate observation from each lot;

^b^ Coefficient of variance of 12 observations at each concentration;

^c^ Expressed as 100x(mean calculated concentration)/(nominal concentration);

^d^ Heamolyzed lot (2% heamolysis);

^e^ Lipemic lot

**Tab. 3.** Absolute matrix effect and recovery of the developed extraction method for amlodipine and valsartan

Analyte	QC Sample	A (%CV)^a^	B (%CV)^a^	C (%CV)^a^	%AME^b^	%Recovery^c^
Amlodipine	LQC	5998 (3.4)	7990 (4.8)	8220 (6.3)	97.2	73.0
	MQC	96333 (4.4)	122985 (2.9)	119986 (1.9)	102.5	80.3
	HQC	151341 (3.5)	178605 (2.4)	182251 (1.9)	98.0	83.0
Valsartan	LQC	16255 (4.3)	20404 (3.8)	21014 (5.5)	100.4	77.4
	MQC	675463 (4.2)	8072754 (2.7)	8129662 (3.2)	99.3	83.1
	HQC	11698961 (4.5)	13515308 (1.9)	13368258 (2.0)	101.1	87.5

A: Mean area response of six replicate samples obtained after extraction;

B: Mean area response of six replicate samples obtained after reconstituting the blank post-extracted samples with aqueous QC samples;

C: Mean area response of six replicate samples prepared in reconstitution solution;

^a^ Coefficient of variation;

^b^ B/Cx100;

^c^ A/Cx100.

Stock solutions of the analyte and ISTD were found to be stable for 7 days at 1–10°C. The percentage stability of amlodipine, valsartan, amlodipine-d4, and valsartan-d9 were 99.9%, 100.3%, 103.3%, and 100.7%, respectively. Working solutions of each analyte were evaluated for 8.67 hr in an ice-cold water bath, under low light conditions, and found to be stable. Analytes were proven to be stable in human K_3_EDTA whole blood for ~2.0 hr and stable in plasma for three freezes-thaw cycles. Benchtop stability for amlodipine and valsartan in human plasma was established for 6.80 hr, autosampler stability was assessed for 73.75 hr at 10°C, and long-term stability was established at -50°C for 129 days. The mean % nominal values of the analytes were found to be within 15% of the predicted concentrations and % CV was less than 15% for the analytes in the stability samples. The % change in analyte concentrations under the stability conditions at the LQC and HQC levels are presented in Table 4. The % difference for all re-injected QC samples was < 8 when compared to the original concentration. No carryover in the matrix was observed for the analytes and internal standards. In dilution integrity, the samples which were diluted by two and four times with the blank matrix were each run in six replicates, which had a precision of < 4% and % nominal within 6% of the actual concentration for two analytes.

**Tab. 4.** Stability of amlodipine and valsartan in different storage conditions (n=4)

Stability	QC Sample	%Change^a^	%Change^b^
Benchtop stability	LQC	0.8	-5.7
(~ 6.80 hr, in ice-cold water bath)	HQC	0.1	1.0

Freeze-thaw stability	LQC	10.1	-12.4
(3 freeze-thaw cycles)	HQC	-0.7	-2.7

Autosampler stability	LQC	1.3	-5.4
(~73.75 hr at 10°C)	HQC	0.6	-1.3

Long-term stability	LQC	-4.0	1.4
(129 days, below -50°C)	QC	0.5	-1.5

^a^ % change in stability samples of amlodipine when compared to comparison samples;

^b^ % change in stability samples of valsartan when compared to comparison samples.

### Method Application

**Fig. 3.**
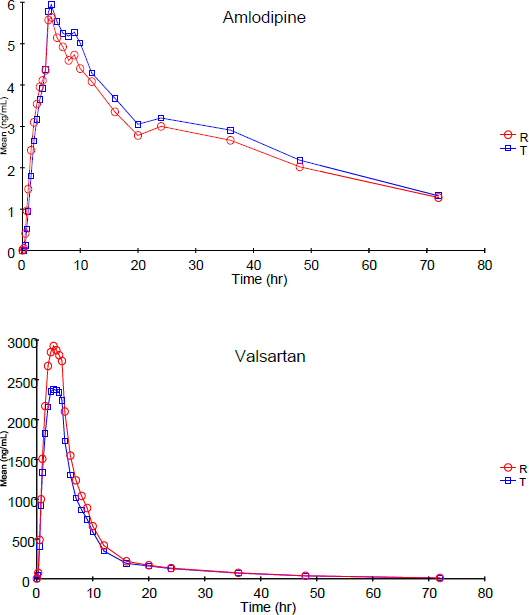
Linear plot of the mean plasma concentration (ng/mL) versus time (hr) of amlodipine and valsartan, respectively (n=42); R: reference drug; T: test drug.

Following analysis, the pharmacokinetic parameters were calculated by non-compartmental modeling of data using WinNonlin professional software (Version 5.0, Pharsight Corp., Mountain View, CA, USA). The peak plasma concentration (C_max_) and time to reach C_max_ (T_max_) were read directly from the data. The total area under the plasma concentration-time curve from time zero to the last measurable concentration (AUC_0→*t*_) was calculated using the linear trapezoidal rule-extrapolation method. Figure 3 shows the linear plot of the mean plasma concentration (ng/mL) versus time (hr) for both analytes. The mean estimates of the pharmacokinetic parameters derived from the plasma concentration profiles are summarized in Table 5. Of the total reanalyzed samples, 205 samples for amlodipine and 178 samples for valsartan met the acceptance criteria, showing good reproducibility of the method.

**Tab. 5.** Pharmacokinetic parameters of amlodipine and valsartan

Parameters	Amlodipine		Valsartan	
	Reference drug	Test drug	Reference drug	Test drug
Tmax (hr)	5.42±1.59	5.61±1.64	3.02±1.01	2.96±1.17
Cmax (ng/mL)	6.05±1.26	6.41±1.35	3527.62±1765.83	2992.51±1360.43
AUC0±→t (hr.ng/mL)	190.10±44.67	202.53±50.43	22892.5±10002.1	19459.7±7389.3

Parameters are the mean (± SD) values of 42 healthy human subjects.

## Conclusion

In this study, a simple, selective, accurate, and reproducible LC-MS/MS method in positive ESI mode was developed and validated for the simultaneous estimation of amlodipine and valsartan in human plasma. The method shows good performance with respect to all the validation parameters tested. In addition, the present method utilizes the solid-phase extraction method and offers high throughput because of a shorter run time. The applicability of the method was demonstrated by the successful completion of a bioequivalence study of the amlodipine and valsartan fixed-dose combination in human subjects.

## Experimental

### Chemicals and Materials

Amlodipine and valsartan were received from Ranbaxy Laboratories Limited, India. Amlodipine-d4 and valsartan-d9 were obtained from Varda Biotech Pvt Ltd., India and TLC PharmaChem., Canada, respectively. Ammonium formate was obtained from Fluka (Buchs, Switzerland). Formic acid was obtained from Fisher Scientific (India). Acetonitrile was obtained from Spectrochem (India). Milli-Q water acquired from the Millipore water purification system (Molsheim Cedex, France) was used in the preparation of solutions. Plasma batches containing K_3_EDTA (salt of ethylenediaminetetraacetic acid tripotassium) as an anticoagulant were obtained from Yash Laboratories, Pune, India.

### LC-MS/MS Instrumentation and Chromatographic Conditions

The instrumentation consisted of a modular HPLC (Shimadzu, Kyoto, Japan) coupled to AB Sciex API-4000 mass spectrometer (Applied Biosystems, Ontario, Canada), equipped with an electrospray ion interface operating in positive mode using nitrogen as the nebulizer, auxiliary, collision, and curtain gas. The HPLC system consisted of two LC-20AD pumps, online DGU-20A3 solvent degasser, a SIL-HTc autosampler, and a CTO-20A column oven. A mobile phase consisting of acetonitrile and 5 mM ammonium formate buffer (80:20, *v/v*) was delivered at a flow rate of 0.8 mL/min through the Luna C18 (2) column in a column oven maintained at 35°C. The chromatograms were acquired using analyst software (version 1.4.1, Applied Biosystems, Ontario, Canada). Calibration curves were constructed using the peak area ratio of the analyte to the internal standard versus the analyte concentration and by applying the weighted least squares regression algorithm.

### Preparation of Stock Solutions, Calibration Standards, and Quality Control Samples

Stock solutions of the analytes and internal standards were prepared in methanol and stored between 1 to 10°C. The concentrations were corrected for purity, moisture content, and actual amount weighed as per their certificate of analysis. Working solutions of each analyte for the calibration curve (CC) standards and quality control samples (QC) were prepared in methanol: water (50:50, v/v) separately. The 1% spikings of these working solutions were done individually in plasma to attain the desirable concentration of each analyte. Each calibration curve consisted of one blank sample, one blank sample fortified with IS, and eight calibration points ranging from 0.302–20.725 ng/mL for amlodipine and from 6.062–18060.792 ng/mL for valsartan. The QC samples spiked independently of the CC standard stock comprised of the limit of quantification quality control (LOQQC), low quality control (LQC), middle quality control (MQC), and high quality control (HQC). Aliquots of the CC and QC standards were stored below -50°C.

### Biological Sample Preparation

To 300 μL of the plasma samples, 50 μL of ISTD (internal standard) dilution (containing approximately 100ng/mL and 1000ng/mL of amlodipine-d4 and valsartan-d9, respectively) was added and the samples were pre-treated with 300 μL of 5% ortho phosphoric acid solution (v/v) and vortexed. The pre-treated samples were loaded onto conditioned cartridges (Oasis, HLB 30 mg/1cc, Waters Corporation, USA) and centrifuged (Eppendorf Refrigerated centrifuge, 5810R) at 1500 rpm for 1 min. Then cartridges were washed with 1 ml of 5% methanol in water (v/v) followed by 1 ml of Milli-Q water. Finally, the elution was carried out with 1 ml methanol. The eluent was evaporated to dryness using the Zymark TurboVap® LV evaporator (caliper, Hopkinton, MA, USA) at 50°C. The dried residue was reconstituted with 300 ul of the mobile phase. Ten uL of each sample was injected into the LC-MS/MS for analysis.

### Method Validation

The developed method was comprehensively validated as per US Food and Drug Administration (USFDA) guidelines and guidance from the European Medical Agency [28, 29]. The method was validated for selectivity, sensitivity, linearity, precision and accuracy, recovery, dilution integrity, matrix effect, re-injection reproducibility, carryover in the matrix, and stability of the analytes during both short-term sample processing and long-term storage.

Selectivity of the method was assessed using eight different lots of human K3EDTA plasma (which included six normal lots, one hemolyzed, and one lipemic) by screening for the responses of the interfering substances at the retention times of the analytes and internal standards. Interference in each plasma batch was compared to six limit of quantification (LOQ) samples, prepared by pooling two normal blank matrix batches with minimal or no peak area response at the retention time of all peaks. Response of the interfering peaks at the retention times of the analyte and internal standard in the blank matrix must be ≤ 20% of the mean peak area response of the analyte in the LOQ samples for the analyte and must be ≤ 5% of the mean peak area response of the internal standard in the LOQ samples for ISTD. The signal-to-noise ratio of each LOQ sample should be at least five times of that of the blank matrix samples.

Four calibration curves were used to demonstrate the linearity of the method. The best-fit curves using weighted linear least square regression analysis were obtained by the peak area ratio of the analyte to internal standard versus analyte concentration. A correlation coefficient r > 0.99 was desirable for all the calibration curves.

The intra- and interday accuracy and precision of the method were determined for the estimation of the analytes in human K_3_EDTA plasma. They were estimated by replicate analysis of precision and accuracy (PA) batches. Each PA batch consisted of CC standards and six replicates of QC at each level. Intraday precision and accuracy were determined by analyzing two PA batches processed on the same day. Interday assay precision and accuracy were determined by analyzing four PA batches processed in the duration of 3 days. An extended PA batch, which stimulates the real time analysis during the estimation of unknown samples, consisting of 45 replicates of QC samples at the LQC, MQC, HQC levels against the CC standards, was processed and analyzed. The analyte peak of the LOQ/LOQQC sample should be identifiable, discrete, and reproducible with a precision (% CV) not greater than 20.0% and accuracy within 20.0%. For standards/QC samples other than the LOQ/LOQQC, accuracy should be within 15% and precision not greater than 15.

Matrix effect (relative) was investigated to ensure that the precision and sensitivity of the method were not compromised by the matrix. In order to estimate it, six lots of plasma matrix including lipemic and hemolyzed were chosen and concentrations equivalent to the LOQQC and HQC levels were spiked in duplicate in each lot. Also, freshly spiked CC standards, six LOQQC samples, and six HQC samples were prepared in pooled plasma and processed. The values of the QC samples were back-calculated against the freshly spiked CC standards. For the calculation of the absolute matrix effect (AME), working solutions of the drug and ISTD were prepared at concentrations representing 100% extraction of the QC samples at low, middle, and high concentrations (aqueous samples). Six aliquots from each of six different batches of the screened blank matrix, including one lipemic and one hemolyzed matrix, were taken and processed without the addition of the internal standard. Two aliquots of each post-extracted blank matrix were reconstituted with solution representing the final extracted concentration of the analytes at each QC level. AME is acceptable if %CV at each QC level is ≤ 15% and the %CV between QC levels is ≤ 15%.


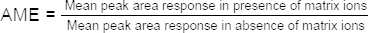


Where AME =1 indicates no matrix effect, AME <1 indicates ion suppression, and AME >1 indicates ion enhancement. Recovery (process efficiency) of the developed extraction method was determined by comparing the mean peak area of the analyte in the processed plasma samples which were pre-spiked with the analytes at the LQC, MQC, HQC levels with the dilution of analytes representing 100% extraction of the QC samples. Similarly, recovery of the ISTD was determined by comparing the mean peak area of ISTD in the extracted MQC samples (n=6) with the dilution representing 100% extraction of the ISTD sample.

Stability of the analytes was evaluated in plasma under different conditions which occurred during incurred sample handling, and analysis was evaluated during the method validation. Stock solution stability was performed by comparing the area response of the analyte and internal standard in the stability sample, with the area response of the sample prepared from fresh stock solutions. The stability of the working solutions of each analyte was evaluated by comparing the peak area response of the stability working solution kept in ice-cold water under low light, with the area response of the freshly prepared working solution. The stability of the spiked human plasma samples stored in an ice-cold water bath under low light (benchtop stability) was evaluated for ~ 7 hr. The autosampler stability was determined by stored reconstituted stability QC samples for ~ 72 hr under autosampler conditions (at 10°C) before being analyzed. The freeze-thaw stability was conducted by comparing the stability samples that had been frozen at -50°C and thawed at room temperature three times. For long-term stability evaluation, the concentrations obtained after 129 days were compared with the initial concentrations. All stability exercises were performed against freshly spiked CS. Stability studies in plasma were performed at the LQC and HQC levels using four replicates at each level. The analyte was considered stable if the % change was less than 15, and was calculated by using the following formula:


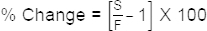


Where, S = mean concentration of stability samples and F= mean concentration of comparison samples.

For sample collection and handling stability, fresh human K_3_EDTA whole blood was spiked with the analytes at the LQC and HQC levels and kept in an ice-cold water bath for ~2.5 hr. After the stability period, fresh blood was spiked again and these samples served as comparison samples. The stability and comparison samples were centrifuged at 4°C and the resulting plasma was collected and processed as per the developed analytical method. The stability was calculated as mentioned above.

Re-injection reproducibility was performed by injecting all QC samples from an accepted precision-accuracy batch. The calculated concentration of the re-injected QC samples was determined against the CC samples from the same precision and accuracy batch which was analyzed 48 hr before. The % difference between the original and re-injected value was calculated by using formula:


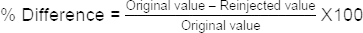


Carryover in the matrix was estimated by injecting duplicates of the LOQ and upper limit of quantification (ULOQ) samples, bracketed with the same processed blank sample. Interferences at the retention time of the analytes and ISTD were evaluated by comparing the difference in area response of the first blank matrix to the second blank sample against their respective mean peak area response of the analytes and ISTD in the processed LOQ samples.

The dilution integrity experiment was performed with an aim to validate the dilution test to be carried out on higher analyte concentrations above the ULOQ, which may be encountered during real time incurred sample analysis. The dilution integrity test was performed by preparing samples at a concentration approximately two times the concentration of 90% of the ULOQ. These samples were diluted to two and four times with the blank matrix to bring the concentration within the calibration curve and then analyze against the fresh CC samples.

### Application to Bioequivalence Study

The method was applied to an open label, balanced, randomized, two-treatment, two-period, two-sequence, single-dose, crossover bioequivalence study comparing the amlodipine besylate 10 mg and valsartan 160 mg fixed-dose combination tablet of Ranbaxy Laboratories Limited, India with Norvasc® 10 mg tablet (containing amlodipine besylate 10 mg) of Pfizer Oy, Finland co-administered with Diovan^®^ 160 mg tablet (containing valsartan 160 mg) of Novartis Finland Oy in healthy, adult human subjects. This study conformed fully to the principles enunciated in the Declaration of Helsinki. The study fully adhered to the principles of good clinical practices outlined in the ethical guidelines for Biomedical Research on Human Participants issued by the Indian Council of Medical Research, New Delhi and the *Guideline for Good Clinical Practice* (ICH E6 Guideline, May 1996). Forty-two healthy subjects from whom prior informed consent was taken were enrolled and the study was approved by the Jamia Hamdard Institutional Review Board, New Delhi, India. Blood samples were collected in vacutainer tubes containing anticoagulant, prior to dosing (pre-dose) and at 0.250, 0.500, 0.750, 1.000, 1.500, 2.000, 2.500, 3.000, 3.500, 4.000, 4.500, 5.000, 6.000, 7.000, 8.000, 9.000, 10.000, 12.000, 16.000, 20.000, 24.000, 36.000, 48.000, and 72.000 hr post-dose in each period. Plasma was separated by centrifugation and the separated plasma samples were stored below -50°C until analysis.

An incurred sample reanalysis (ISR), to show reproducibility of method, was performed by selecting five samples from each subject who completed all periods of the study. In each period, the time point at the Cmax level and the time point from the elimination phase (concentration at LQC level) were selected which cover the complete profile obtained from a subject. Thus, the ISR was performed on 208 and 178 sample points from 42 different subjects for amlodipine and valsartan, respectively. For each analyte, the difference between the original and reanalyzed values should be within 20% for at least 67% of the total samples reanalyzed [30].


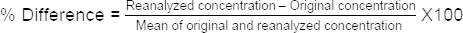


## References

[1] Miura S Saku K Efficacy and safety of angiotensin II type 1 receptor blocker/calcium channel blocker combination therapy for hypertension: focus on a single-pill fixed-dose combination of valsartan and amlodipine. J Int Med Res. 2012; 40: 1–9. http://dx.doi.org/10.1177/147323001204000101 22429340 10.1177/147323001204000101

[2] Gradman A Basile J Carter B Bakris G Combination therapy in hypertension. J Clin Hypertens. 2011; 13: 146–154. http://dx.doi.org/10.1111/j.1751-7176.2010.00397.x 10.1111/j.1751-7176.2010.00397.x PMC8673364 21366845

[3] Shakil A Fixed-dose Combination therapy in Hypertension: Focus on Fixed-dose combination of amlodipine and valsartan. Clin Med Insights Ther. 2009; 1: 1521–1529.

[4] Murdoch D Heel R Amlodipine: A review of its pharmacodynamic and pharmacokinetic properties and therapeutic use in cardiovascular disease. Drugs. 1991; 41: 478–505. http://dx.doi.org/10.2165/00003495-199141030-00009 1711448 10.2165/00003495-199141030-00009

[5] Criscione L Gasparo M Buhlmayer P Whitebread S Ramjoue H Wood J Pharmacological profile of valsartan: A potent, orally active, nonpeptide antagonist of the angiotensin II AT1-receptor subtype. Br J Pharmacol. 1993; 110: 761–767. http://dx.doi.org/10.1111/j.1476-5381.1993.tb13877.x 8242249 10.1111/j.1476-5381.1993.tb13877.x PMC2175903

[6] Shimooka K Sawada Y Tatematsu H Analysis of amlodipine in serum by a sensitive high-performance liquid chromatographic method with amperometric detection. J Pharm Biomed Anal. 1989; 7: 1267–1272. http://dx.doi.org/10.1016/0731-7085(89)80130-X 2535105 10.1016/0731-7085(89)80130-x

[7] Monkman S Ellis J Cholerton S Thomason J Seymour R Automated gas chromatographic assay for amlodipine in plasma and gingival crevicular fluid. J Chromatogr B. 1996; 678: 360–364. http://dx.doi.org/10.1016/0378-4347(95)00526-9 10.1016/0378-4347(95)00526-9 8738044

[8] Maurer H Arlt J Screening procedure for detection of dihydropyridine calcium channel blocker metabolites in urine as part of a systematic toxicological analysis procedure for acidic compounds by gas chromatography-mass spectrometry after extractive methylation. J Anal Toxicol. 1999; 23: 73–80. http://dx.doi.org/10.1093/jat/23.2.73 10192408 10.1093/jat/23.2.73

[9] Tatar S Atmaca S Determination of amlodipine in human plasma by high-performance liquid chromatography with fluorescence detection. J Chromatogr B. 2001; 758: 305–310. http://dx.doi.org/10.1016/S0378-4347(01)00197-9 10.1016/s0378-4347(01)00197-9 11486841

[10] Feng Y Zhang L Shen Z Pan F Zhang Z Analysis of amlodipine in human plasma by liquid chromatography-mass spectrometry. J Chromatogr Sci. 2002; 40: 49–53. http://dx.doi.org/10.1093/chromsci/40.1.49 11866387

[11] Bahrami G Mirzaeei S Simple and rapid HPLC method for determination of amlodipine in human serum with fluorescence detection and its use in pharmacokinetic studies. J Pharm Biomed Anal. 2004; 36: 163–168. http://dx.doi.org/10.1016/j.jpba.2004.05.016 15351061 10.1016/j.jpba.2004.05.016

[12] Nirogi R Kandikere V Mudigonda K Sensitive and rapid liquid chromatography/tandem mass spectrometry assay for the quantification of amlodipine in human plasma. Biomed Chromatogr. 2006; 20: 833–842. http://dx.doi.org/10.1002/bmc.600 16397912 10.1002/bmc.600

[13] Bhatt J Singh S Subbaiah G A rapid and sensitive liquid chromatography-tandem mass spectrometry (LC-MS/MS) method for the estimation of amlodipine in human plasma. Biomed Chromatogr. 2007; 21: 169–175. http://dx.doi.org/10.1002/bmc.730 17221911 10.1002/bmc.730

[14] Ma Y Qin F Sun X Determination and pharmacokinetic study of amlodipine in human plasma by ultra performance liquid chromatography electrospray ionization mass spectrometry. J Pharm Biomed Anal. 2007; 43: 1540–1545. http://dx.doi.org/10.1016/j.jpba.2006.11.015 17174058 10.1016/j.jpba.2006.11.015

[15] Suchanova B Kostiainenc R Ketola R Characterization of the in vitro metabolic profile of amlodipine in rat using liquid chromatography-mass spectrometry. Eur J Pharm Sci. 2008; 33: 91–99. http://dx.doi.org/10.1016/j.ejps.2007.10.003 18055188 10.1016/j.ejps.2007.10.003

[16] Gonzalez L Lopez J Alonso R Jimenez R Fast screening method for the determination of angiotensin II receptor antagonists in human plasma by high-performance liquid chromatography with fluorimetric detection. J Chromatogr A. 2002; 9499: 49–60. http://dx.doi.org/10.1016/S0021-9673(01)01496-0 11999756 10.1016/s0021-9673(01)01496-0

[17] Macek J Klima J Ptacek P Rapid determination of valsartan in human plasma by protein precipitation and high-performance liquid chromatography. J Chromatogr B. 2006; 832: 169–172. http://dx.doi.org/10.1016/j.jchromb.2005.12.035 10.1016/j.jchromb.2005.12.035 16426905

[18] Iriarte G Ferreiros N Ibarrondo I Alonso R Maguregui M Jimenez R Biovalidation of an SPE-HPLC-UV-fluorescence method for the determination of valsartan and its metabolite valeryl-4-hydroxy-valsartan in human plasma. J Sep Sci. 2007; 304: 2231–2240. http://dx.doi.org/10.1002/jssc.200700033 17694512 10.1002/jssc.200700033

[19] Koseki N Kawashita H Hara H Niina M Tanaka M Kawai R Nagae Y Masuda N Development and validation of a method for quantitative determination of valsartan in human plasma by liquid chromatography-tandem mass spectrometry. J Pharm Biomed Anal. 2007; 43: 1769–1774. http://dx.doi.org/10.1016/j.jpba.2006.12.030 17289324 10.1016/j.jpba.2006.12.030

[20] Alnajjar AO Validation of a capillary electrophoresis method for the simultaneous determination of amlodipine besylate and valsartan in pharmaceuticals and human plasma. JAOAC Int. 2011; 94: 498–502. http://www.ncbi.nlm.nih.gov/pubmed/21563683 21563683

[21] Nashwah GM Simultaneous determination of amlodipine and valsartan. Anal Chem Ins. 2011; 6: 53–59. http://dx.doi.org/10.4137/ACI.S7282 10.4137/ACI.S7282 PMC3169342 21918600

[22] El-Gizawy SM Abdelmageed OH Omar MA Deryea SM Abdel-Megied AM Development and Validation of HPLC method for simultaneous determination of amlodipine, valsartan, hydrochlorothiazide in dosage form and spiked human plasma. Am J Anal Chem. 2012; 3: 422–430 http://dx.doi.org/10.4236/ajac.2012.36055

[23] Sharma R Pancholi S Simple RP-HPLC method for determination of triple drug combination of valsartan, amlodipine and hydrochlorothiazide in human plasma. Acta Pharm. 2012; 62: 45–58. http://dx.doi.org/10.2478/v10007-012-0004-3 22472448 10.2478/v10007-012-0004-3

[24] Kepekci Tekkeli SE Development of an HPLC-UV Method for the Analysis of Drugs Used for Combined Hypertension Therapy in Pharmaceutical Preparations and Human Plasma. J Anal Methods Chem. 2013; 179627. http://dx.doi.org/10.1155/2013/179627 10.1155/2013/179627 PMC3619551 23634320

[25] Kristoffersen L Oiestad E Opdal M Krogh M Lundanes E Christophersen A Simultaneous determination of 6 beta-blockers, 3 calcium-channel antagonists, 4 angiotensin-II antagonists and 1 antiarrhytmic drug in post-mortem whole blood by automated solid phase extraction and liquid chromatography mass spectrometry Method development and robustness testing by experimental design. J Chromatogr B. 2007; 850: 147–160. http://dx.doi.org/10.1016/j.jchromb.2006.11.030 10.1016/j.jchromb.2006.11.030 17175206

[26] Gonzalez O Alonso R Ferreiros N Weinmann W Zimmermann R Dresen S Development of an LC-MS/MS method for the quantitation of 55 compounds prescribed in combined cardiovascular therapy. J Chromatogr B. 2011; 870: 243–252. http://dx.doi.org/10.1016/j.jchromb.2010.12.007 10.1016/j.jchromb.2010.12.007 21190906

[27] Ramani A Sengupta P Mullangi R Development and validation of a highly sensitive and robust LC-ESI-MS/MS method for simultaneous quantitation of simvastatin acid, amlodipine and valsartan in human plasma: application to a clinical pharmacokinetic study. Biomed Chromatogr. 2009; 23: 615–622. http://dx.doi.org/10.1002/bmc.1161 19277959 10.1002/bmc.1161

[28] Guidance for Industry: Bioanalytical Method Validation. US Department of Health and Human Services, Food and Drug Administration, Center for Drug Evaluation and Research, Rockville, MD, USA, 2001.

[29] Guideline on bioanalytical method validation. European Medical Agency, Committee for Medicinal Products for Human Use, 7 Westferry Circus, Canary Wharf, UK, 2011.

[30] Viswanathan C Bansal S Booth B Workshop/conference report—quantitative bioanalytical methods validation and implementation: best practices for chromatographic and ligand binding assays. AAPS J. 2007; 9: E30–E42. http://dx.doi.org/10.1208/aapsj0901004 10.1007/s11095-007-9291-7 17458684

